# A facile, stereoselective, one-pot synthesis of resveratrol derivatives

**DOI:** 10.1186/s13065-015-0102-7

**Published:** 2015-05-20

**Authors:** Vishal C Birar, Angela N Sheerin, Jana Milkovicova, Richard G A Faragher, Elizabeth L Ostler

**Affiliations:** Ageing Research Group, School of Pharmacy and Biomolecular Sciences, University of Brighton, Moulsecoomb, BN2 4GJ Brighton, UK

**Keywords:** Resveratrol, Stilbene, Ageing

## Abstract

**Background:**

Compounds based on *trans-*1,2-diphenylethene are the subject of intense interest both for their optical properties and as potential leads for drug discovery, as a consequence of their anticancer, anti-inflammatory and antioxidant properties. Perhaps the best known of these is *trans*-3,5,4′-trihydroxystilbene (resveratrol), that has been identified as a promising lead in the search for anti-ageing therapeutics.

**Results:**

We report here a new, convenient, one-pot stereo-selective synthesis of resveratrol and other *trans*-stilbene derivatives. A wide range of known and novel “Resveralogues” were synthesised by using this simple protocol, including examples with electron donating and electron withdrawing substituents, in uniformly high yield. The structures of all compounds were confirmed by standard methods including ^1^H and ^13^C NMR, IR and High Resolution Mass spectroscopy.

**Conclusions:**

We have established a simple and convenient protocol for resveralogue synthesis. It is readily scalable, and sufficiently robust and simple for ready use in automated synthesis or for library development of resveralogues. This supersedes previously reported synthetic methods that required inert conditions, extensive purification and/or costly reagents.

**Graphical abstract:**
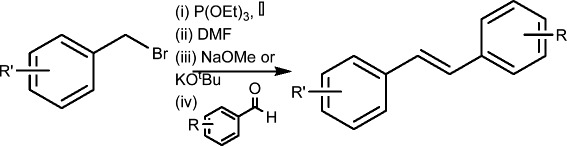
One-pot preparation of diverse Resveralogues - high yields of product with minimal purification.

## Background

*trans*-1,2-Diphenylethene is the basic structural unit in a wide variety of naturally-occurring molecules. These, and synthetic analogues, have been deployed in photochemical dyes and fluorescent whitening agents [1], polymeric materials [2, 3], and are the subject of intense interest as potential leads for drug discovery as a consequence of their anticancer [4, 5] anti-inflammatory [6] and antioxidant properties [7]. Perhaps the best known of these is *trans*-3,5,4′-trihydroxystilbene (resveratrol), which has shown potential clinical value as a dietary restriction mimetic. Such mimetics are thought to slow the rate of deleterious processes associated with ageing and thus have the potential to prevent, or even remediate, multiple age-associated degenerative pathologies, including cognitive impairment, arthritis, cardiovascular disease and immune dysfunction [8, 9]. *trans*-Stilbenes thus represent attractive scaffolds for future compound development. However, resveratrol itself has been shown to have a range of activities, some of which may actually be detrimental to health. Equally, resveratrol is very limited in its bioavailability and, taken together, these issues leave uncertainties about its likely *in vivo* modes of action and consequent clinical utility. There is therefore a need for simple and versatile syntheses of a wide variety of structural analogues of resveratrol, or resveralogues, in order to facilitate detailed investigation of stilbenoid structure-activity relationships, and to allow development of potential therapeutic compounds with improved bioavailability.

*tran*s-Stilbene derivatives are generally synthesised utilising Wittig or Horner- Wadsworth- Emmons (HWE) reactions and through catalytic methods such as Heck, Suzuki and Negishi coupling reactions or through the use of organozinc reagents [10]. Many of the examples in the literature suffer from incomplete conversion and low yields, or poor stereoselectivity. Of the existing methods, three have been previously modified to one-pot syntheses. These utilised the oxidative Wittig-Heck reaction [11, 12] two sequential Heck-type reactions of aryl bromide [13] and organozinc reagents [10]. Although some of these protocols provide good yields, they tend to require costly organometallic catalysts, inert reaction conditions, large excesses of reagents [13], long reaction times (>40 h in some instances) and/or complex solvent mixtures. We present here the first example of a robust, one-pot, readily scalable synthesis of diverse *trans*-stilbene derivatives and resveralogues.

## Results and discussion

**Scheme 1:**
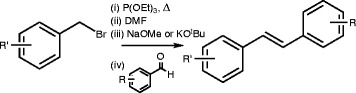
One-pot synthesis of resveralogues

We have developed a simple and convenient one-pot method using sequential Michaelis-Arbuzov rearrangement and HWE chemistry. The overall reaction scheme is shown below (Scheme 1).

Our synthetic protocol reduces both reaction time and reagent usage, by removing the need for isolation and purification both at the intermediate step and in the final product. A wide variety of analogues can conveniently be prepared from existing readily available benzyl bromides and benzaldehyde derivatives. We here describe the preparations of a range (1–16 below) of examples of resveralogues including several novel compounds (2, 4, 6, 8, 15 and 16), in good yield and purity, with minimal waste and manipulative steps.

Our method is facile, versatile and cost effective. We have applied this protocol to the synthesis of a range of differently substituted derivatives and in each case the required *trans*-stilbene was produced in good yield either without purification or through simple recrystallisation. The compounds were characterised by ^1^H NMR, ^13^C NMR, IR, high resolution mass spectroscopy and melting point, and where possible confirmed by comparison to literature values. The characteristic pattern of *trans*-coupling constant (*J*) value (more than 15 MHz) was observed in the ^1^H NMR spectra of all compounds synthesised, and indicated >95 % *trans* selectivity, in that signals from the alternate isomer were not observed.

### Experimental

We selected the Michaelis-Arbuzov rearrangement as the most versatile method available for preparation of phosphonate esters [14, 15]. In this step, we reacted the starting substituted benzyl bromides with a single molar equivalent of triethylphosphite in the absence of solvent with heating to 150 °C. This abrogates the need for the large excesses of triethylphosphite utilised in earlier reports and also avoids potential by-product formed as a consequence of the reaction of ethyl bromide with the excess triethylphosphite. It is usual, at this stage, to isolate and then purify the diethylphosphonate intermediate via column chromatography or distillation [16]. In our protocol, however, we continue directly to the HWE reaction, giving the desired stilbenes in high yield and purity. In most cases the conversion to *trans*-stilbene is complete after 12 h of heating to reflux, and the product can be crystallised directly from the reaction mixture by simple addition of ice and a small quantity of methanol. Where necessary, further purification can be achieved by recrystallisation from ethanol. The choice of base for the HWE reaction is important to the success of this one pot synthesis. Our initial efforts focused on the reaction of 2- and 4- substituted phosphonates to give the relevant stilbenes and these proceed readily to completion with the use of 1.2 equivalents of potassium *t-*butoxide as base. However, when deactivating *meta* and/or electron donating substituents are present potassium *t-*butoxide is unsuccessful and a stronger base must be used. We found that 1.1 equivalents of sodium methoxide was sufficient to ensure the reaction proceeded successfully when such groups were present, although in some cases the yield was a little reduced.

## Conclusions

Our one-pot protocol is very efficient and stereoselective for the synthesis of a wide range of resveralogues. The utility and applicability of this method is enhanced by its simple work up procedure, rendering it also suitable for use in automated synthesis.

## Methods

### General

The starting materials and solvent, reagents were obtained commercially and used directly without purification. The NMR spectra of compounds were recorded on Brüker FT-NMR 400Hz spectrometer in CDCl_3_ using tetramethylsilane (TMS) as an internal standard. The δ values represent chemical shifts reported in parts per million (ppm) and coupling constant (*J*) values are in Hz. Assignments correspond to R and R’ as per Table 1, numbering each ring from the carbon closest to the central double bond. ^13^C NMR spectra were definitively assigned with reference to HSQC correlation spectra (not presented). ESI-MS and ESI-HRMS were recorded on a Brüker MicroTOF instrument. Melting points were recorded on an Electrothermal melting point apparatus and are uncorrected. Flash chromatography was conducted by using Silica size (100–200 mesh). Thin layer chromatography was performed on TLC Silica Gel 60 F254 (Merck).

**Table 1:** Preparation of resveralogues

	R’^†^	R^†^	Yield (%)
1	2-CN	3,5-dimethoxy	71
2	3-Cl	4-N(Me)_2_	79
3	2-NO_2_	3,5-dimethoxy	80
4	2-F	3,5-dimethoxy	74
5	2-NO_2_	4-OMe	79
6	3-Cl	3,5-dimethoxy	68
7	3-CF_3_	4-N(Me)_2_	74
8	3,5-dimethyl	3,5-dimethoxy	81
9	2,4-difluoro	3,5-dimethoxy	74
10	4-Me	3,5-dimethoxy	84
11	4-CN	3,5-dimethoxy	80
12	4-OMe	3,5-dimethoxy	60
13	4-COOMe	3,5-dimethoxy	59
14	4-NO_2_	3,5-dimethoxy	90
15	2,6-difluoro	3,5-dimethoxy	78
16	2,6-dichloro	3,5-dimethoxy	79

^†^R’ being the substituent(s) of the benzyl bromide starting material and R, the aldehyde

### General procedure for synthesis of substituted *trans*-stilbenes

To a two-necked oven dried round-bottomed flask, the required benzyl bromide (500 mg, 1 equivalent) and triethylphosphite (1 equivalent) were added with N_2_ purging. This reaction mixture was heated at 150 °C for 4-5 h. The reaction was monitored by thin layer chromatography (eluent 10:90::ethyl acetate: petroleum ether). After completion, the reaction mixture was cooled to room temperature and then diluted tenfold with *N,N*-dimethylformamide. Base (for procedure A, KOtBu (1.1 equivalents) and for procedure B, NaOMe (1.2 equivalents)) was added and stirred at room temperature for 10 min. Aldehyde (0.8 equivalents) was then added to the reaction mixture which was then stirred at room temperature for a further hour. After this time it was heated to reflux for 12 h. The reaction was cooled to room temperature and quenched by adding ice and a small amount of methanol. The resulting precipitate was then filtered and dried to give the crude stilbene as title product. The crude product was recrystallised from ethanol or ethyl acetate to give fine and pure crystals.

#### (*E*)-2-(3,**5**-dimethoxystyryl)benzonitrile (1)

Procedure A gave 478 mg white solid (71 %), m.p. = 72–73 °C, Rf (10:90::ethyl acetate:petroleum ether) = 0.27, IR ν (cm^-1^) = 3068, 2948, 2838, 2219, 1588, 1425, 1352, 1152, 1056, 958, 755, ^1^H NMR (400 MHz, CDCl_3_) δ (ppm) = 3.84 (6H, s, -OMe), 6.45 (1H, t, *J*2Hz, H4), 6.72 (2H, d, *J*2Hz, H2, H6), 7.19 (1H, d, *J*16Hz, -C = C-H), 7.33 (1H, td, *J*7.6,1.1Hz, H4′), 7.41 (1H, d, *J*16Hz, -C = C-H), 7.57 (1H, td, *J*7.6, 1Hz, H5′), 7.65 (1H, dd, *J*8.0, 1Hz, H6′), 7.78 (1H, d, *J*8.0Hz, H3′). ^13^C NMR (100 MHz, CDCl_3_) δ (ppm) = 55.4 (OMe), 101.2 (C4), 105.2 (C2, C6), 111.3 (C2′), 117.4 (CN), 124.6 (C = C), 125.4 (C3′), 127.6 (C4′), 132.7 (C5′), 133.2 (C6′), 133.5 (C = C), 138.2 (C1′), 140.4 (C1), 161.1 (C3, C5). HRMS *m/z* calculated 288.09950 [(M + Na)]^+^ and 533.20978 [2 M + Na]^+^, found 288.09945 and 553.20737.

#### (*E*)-4-(3-chlorostyryl)-*N,N*-dimethylaniline (2)

Procedure B gave 495 mg white solid (79 %). m.p. = 138–139 °C. Rf (10:90::ethyl acetate:petroleum ether) = 0.56, IR ν (cm^-1^) = 3031, 2893, 1608, 1582, 1065, 965, 808, 730, ^1^H NMR (400 MHz, CDCl_3_) δ (ppm) = 2.98 (6H, s, -N(CH_3_)_2_), 6.71 (distorted d, *J*8.8 Hz, H3, H5), 6.83 (1H, d, *J*16.4Hz, -C = C-H), 7.02 (1H, d, *J*16.4Hz, -C = C-H), 7.15 (1H, ddd, J8.0, 2.0,1.2, H6′), 7.24 (1H, t, *J*8Hz, H5′), 7.32 (1H, distorted d, *J*7.6Hz, H4′), 7.40 (2H, distorted d, *J*8.8Hz, H2, H6), 7.45 (1H, t, *J*3.5Hz, H2′), ^13^C NMR (100 MHz, CDCl_3_) δ (ppm) = 40.4 (N(CH_3_)_2_), 112.4 (C3,C5), 122.8 (C = C), 124.2 (C4′), 125.4 (C1), 125.8 (C2′), 126.5 (C6′), 127.8 (C2, C6), 129.7 (C5′), 130.3 (C = C), 134.5 (C1′), 140.2 (C3′), 150.4 (C4). HRMS (ESI) calculated *m/z* 258.10440 [(M + H)]^+^, found 258.09288.

#### (*E*)-1,3-dimethoxy-5-(2-nitrostyryl)benzene (3)

Procedure A gave 528 mg yellow solid (80 %), m.p. = 72–73 °C, Rf (10:90::ethyl acetate:petroleum ether) = 0.27, IR ν (cm^-1^) = 3004, 2940, 2838, 1599, 1516, 1342, 1205, 1154, 1054, 951, 819, 744, 670, ^1^H NMR (400 MHz, CDCl_3_) δ (ppm) = 3.83 (6H, s, -OMe), 6.44 (1H, t, *J*2.4Hz, H4), 6.68 (2H, d, *J*2.4Hz, H2, H6), 7.00 (1H, d, *J*16.4Hz, H-C = C-), 7.40 (1H, td, *J*8.4,1.3Hz, H4′), 7.59 (1H, d, *J*16Hz, H-C = C-), 7.59 (1H, t, *J*7.4Hz, H5′), 7.74 (d, *J*7.6Hz, H6′), 7.96 (1H, dd, *J*8.1,1.1Hz, H3′), ^13^C NMR (100 MHz, CDCl_3_) δ (ppm) = 55.4 (OMe), 100.9 (C4), 105.2 (C2, C6), 124.0 (C = C), 124.8 (C3′), 128.0 (C4′), 128.2 (C6′), 132.9 (C5′), 133.1 (C = C), 133.9 (C1′), 138.5 (C1), 148.1 (C2′), 161.1 (C3, C5). HRMS calculated *m/z* 308.08933 [(M + Na)]^+^, found 308.10436.

#### (*E*)-1-(2-fluorostyryl)-3,5-dimethoxybenzene (4)

Procedure B gave 505 mg shiny white crystals (74 %), m.p. = 62 °C, Rf (10:90::ethyl acetate:petroleum ether) = 0.64, IR ν (cm^-1^) = 3001, 2997, 2954, 1590, 1488, 1357, 1152, 1053, 966, 825, 677, ^1^H NMR (400 MHz, CDCl_3_) δ (ppm) = 3.83 (6H, s, -OMe), 6.41 (1H, t, *J*2.2 Hz, H4), 6.69 (d, *J*2.2Hz, H2, H6), 7.15-7.08 (3H, m (includes d, *J*16.4Hz), H3′, H6′, -C = C-H), 7.26-7.22 (2H, m (includes d, *J*16.4Hz), H5′, -C = C-H), 7.59 (1H, td, *J* = 7.7,1.6Hz, H4′), ^13^C NMR (100 MHz, CDCl_3_) δ (ppm) = 55.4 (-OMe), 100.4 (C4), 104.8 (C2, C6), 115.8 (C3′, *J*_*C-F*_ 21.92Hz), 121.5 (C = C, *J*_*C-F*_ 3.1Hz), 124.2 (C6′, *J*_*C-F*_ 3.1Hz), 125.1 (C1′, *J*_*C-F*_ 11.7Hz), 127.2 (C4′, *J*_*C-F*_ 3.5Hz), 128.9 (C5′, *J*_*C-F*_ 8.5Hz), 131.0 (C = C, *J*_*C-F*_ 4.68Hz), 139.3 (C1), 161.0 (C3, C5), 160.5 (C2′, *J*_*C-F*_ 247.9Hz). HRMS calculated *m/z* 281.09483 [(M + Na)]^+^, found 281.07161.

#### (*E*)-1-(4-methoxystyryl)-2-nitrobenzene (5)

Procedure A gave 466 mg yellow solid (79 %), m.p. = 66–69 °C (literature m.p. = 69.9 °C, [17]), Rf (10:90::ethyl acetate:petroleum ether) = 0.64, IR ν (cm^-1^) = 3081, 2977, 2938, 1600, 1505, 1422, 1337, 1250, 1054, 968, 822, 768, ^1^H NMR (400 MHz, CDCl_3_) δ (ppm) = 3.82 (3H, s, OMe), 6.90 (2H, dd, *J*8.8, 2Hz, H2, H6), 7.04 (1H, d, *J*16Hz, -C = C-H), 7.34 (td, *J*8.3,1.3Hz, H4′), 7.34–7.48 (3H, m (includes d, *J*16Hz), H3, H5, -C = C-H), 7.55 (1H, td, J7.4, 0.9Hz, H5′), 7.72 (1H, dd, *J*7.8, 0.9Hz, H6′), 7.91 (1H, dd, *J*8.2, 1.2Hz, H3′), ^13^C NMR (100 MHz, CDCl_3_) δ (ppm) = 55.4 (OMe), 114.3 (C2, C6), 121.1 (C = C), 124.8 (C3′), 127.5 (C4′) 127.9 (C6′), 128.5 (C3, C5), 129.3 (C1′), 133.0 (C1), 133.3 (C5′), 133.5 (C = C), 147.9 (C2′), 160.1 (C4). HRMS calculated *m/z* 278.07876 [(M + Na)]^+^, found 278.07100.

#### (*E*)-1-(3-chlorostyryl)-3,5-dimethoxybenzene (6)

Procedure B gave, after purification, 454 mg colourless liquid (68 %), b.p. >300 °C, Rf (10:90::ethyl acetate:petroleum ether) = 0.47, IR ν (cm^-1^) = 3030, 2995, 2898, 1603, 1542, 1357, 1205, 1053, 968, 825, 677, ^1^H NMR (400 MHz, CDCl_3_) δ (ppm) = 3.83 (6H, s, OMe), 6.41 (1H, t, *J*2.2Hz, H4), 6.66 (2H, d, *J*2.2Hz, H2, H6), 6.98 (1H, distorted d, *J*16.2Hz, -C = C-H), 7.03 (1H, distorted d, *J*16.2Hz, -C = C-H), 7.22 (1H, dt, *J*8.4,1.2Hz H6′), 7.26 (1H, dd, *J*8.8, 7.6Hz, H5′), 7.35 (dt, *J*7.5, 1.2Hz, H4′), 7.49 (1H, t, *J*1.6Hz, H2′), ^13^C NMR (100 MHz, CDCl_3_) δ (ppm) = 55.4 (OMe), 100.4 (C4), 104.8 (C2, C6), 124.8 (C4′), 126.4 (C2′), 127.6 (C6′), 127.7 (C = C), 129.9 (C5′), 130.2 (C = C), 134.7 (C1′), 138.84 (C3′), 139.08 (C1), 161.06 (C3, C5). HRMS calculated *m/z* 297.06528 [(M + Na)]^+^ and 571.14134 [(2 M + Na)]^+^, found 297.06374 and 571.13963.

#### (*E*)-*N,N*-dimethyl-4-(3-(trifluoromethyl)styryl)aniline (7)

Procedure B gave 450 mg light green crystals (74 %), m.p. = 146 °C, Rf (10:90::ethyl acetate:petroleum ether) = 0.56, IR ν (cm^-1^) = 3026, 2810, 1601, 1521, 1327, 1115, 1071, 965, ^1^H NMR (400 MHz, CDCl_3_) δ (ppm) = 2.99 (6H, s, -N(CH_3_)_2_), 6.72 (2H, dd, *J*7.2, 2.0Hz, H2, H6), 6.91 (1H, d, *J*16.0Hz, -C = C-H), 7.10 (1H, d, *J*16.0Hz, -C = C-H), 7.40–7.44 (4H, m, H4′, H6′, H3, H5), 7.62 (1H, distorted t, H5′), 7.70 (1H, s, H2′), ^13^C NMR (100 MHz, CDCl_3_) δ (ppm) = 40.4 (N(CH_3_)_2_), 112.4 (C2, C6), 122.5 (C2′, *J*_*C-F*_ 3Hz), 122.7 (C = C), 123.0 (C6′, *J*_*C-F*_ 3Hz), 125.0 (C3′), 125.6 (C1′), 127.84 (C3, C5), 128.94 (C5′), 128.99 (C4′), 130.7 (C = C), 131.0 (CF_3_, *J*_*C-F*_ 32Hz), 139.1 (C1), 150.5 (C4). HRMS calculated *m/z* 292.13076 [(M + H)]^+^, found 292.13062.

#### (*E*)-1-(3,5-dimethoxystyryl)-3,5-dimethylbenzene (8)

Procedure B gave, after purification, 545 mg white solid (81 %), m.p. = 66–68 °C, Rf (10:90::ethyl acetate:petroleum ether) = 0.47, IR ν (cm^-1^) = 3256, 3009, 2923, 1598, 1463, 1326, 1164, 1071, 962, 839, 690, ^1^H NMR (400 MHz, d_6_-DMSO) δ (ppm) = 2.29 (6H, s, CH_3_), 3.78 (6H, s, OMe), 6.40 (1H, t, *J*2.4Hz, H4), 6.75 (2H, d, *J*2.4Hz, H2, H6), 6.91 (broad s, H4′), 7.11 (1H, d, *J*16.4Hz, C = C-H), 7.18 (1H, d, *J*16.4Hz, C = C-H), 7.25 (broad s, H2′, H6′), ^13^C NMR (100 MHz, d_6_-DMSO) δ (ppm) = 20.9 (Me), 55.1 (OMe), 99.8 (C4), 104.3 (C2, C6), 124.3 (C2′, C6′), 128.0 (C = C), 129.0 (C = C), 129.2 (C4′), 136.7 (C1′), 137.5 (C3′, C5′), 139.1 (C1), 160.6 (C3, C5). HRMS calculated *m/z* 291.13555 [(M + Na)]^+^, found 291.13523.

#### (*E*)-1-(3,5-dimethoxystyryl)-2,4-difluorobenzene (9)

Procedure B gave 494 mg white crystals (74 %), m.p. = 72–74 °C (literature m.p. = 78 °C, [18]), Rf (10:90::ethyl acetate:petroleum ether) = 0.64, IR ν (cm^-1^) = 3065, 2948, 2841, 1589, 1460, 1281, 1152, 1054, 965, 813, 676, ^1^H NMR (400 MHz, CDCl_3_) δ (ppm) = 3.83 (6H, s, OMe), 6.42 (1H, t, *J*2.4Hz, H4), 6.67 (2H, d, *J*2.4Hz, H2, H6), 6.80–6.91 (2H, m, H5′ and H6′), 7.02 (1H, d, *J*16.4Hz, -C = C-H), 7.16 (1H, d, *J*16.4Hz, -C = C-H), 7.55 (1H, distorted dd, *J*_HF_15.8, 8.4Hz, H3′), ^13^C NMR (100 MHz, CDCl_3_) δ (ppm) = 55.4 (OMe), 100.4 (C4), 104.2 (C6′, *J*_*C-F*_25.6Hz), 104.7 (C2, C6), 111.7 (C5′, *J*_*C-F*_21.31 and 3.63Hz), 120.5 (C = C), 121.5 (C1′, *J*_*C-F*_8.2 Hz), 128.0 (C3′, *J*_*C-F*_5.17Hz), 130.7 (C = C, *J*_*C-F*_2.3Hz), 139.1 (C1), 160.4 (C4′_,_*J*_*C-F*_250.6, 11.6 Hz), 161.0 (C3, C5), 162.2 (C2′, *J*_*C-F*_248.3, 11.8Hz). HRMS calculated *m/z* 299.08541 [(M + Na)]^+^ and 575.18159 [(2 M + Na)]^+^, found 299.10167 and 575.20262.

#### (*E*)-1,3-dimethoxy-5-(4-methylstyryl)benzene (10)

Procedure A gave 577 mg white crystals (84 %), m.p. = 56–57 °C (literature m.p. = 50–52 °C, [19]), Rf (10:90::ethyl acetate:petroleum ether) = 0.54, IR ν (cm^-1^) = 3029, 2961, 2937, 1585, 1450, 1290, 1148, 955, 831, 754, ^1^H NMR (400 MHz, CDCl_3_) δ (ppm) = 2.36 (3H, s, Me), 3.83 (6H, s, OMe), 6.39 (1H, t, *J*2.2Hz, H4), 6.66 (2H, d, *J*2.2Hz, H2, H6), 7.02 (1H, d, *J*16.2Hz, C = C-H), 7.06 (1H, d, *J*16.2Hz, C = C-H), 7.16 (2H, d, *J*7.9Hz, H2′, H6′), 7.40 (2H, d, *J*8.0Hz, H3′, H5′), ^13^C NMR (100 MHz, CDCl_3_) δ (ppm) = 21.2 (Me), 55.4 (OMe), 99.9 (C4), 104.5 (C2, C6), 126.5 (C3′, C5′), 127.7 (C = C), 129.9 (C = C), 129.4 (C2′, C6′), 134.4 (C1′), 137.7 (C4′), 139.6 (C1), 161.01 (C3, C5). HRMS calculated *m/z* 277.11990 [(M + Na)]^+^, found 277.12461.

#### (*E*)-4-(3,5-dimethoxystyryl)benzonitrile (11)

Procedure A gave 541 mg white solid (80 %), m.p. = 105–107 °C (literature m.p. = 106–107 °C, [20]), Rf (10:90::ethyl acetate:petroleum ether) = 0.54, IR ν (cm^-1^) = 2959, 2937, 2841, 2220, 1587, 1340, 1208, 1169, 1066, 948, 833, 679, ^1^H NMR (400 MHz, CDCl_3_) δ (ppm) = 3.84 (6H, s, OMe), 6.45 (1H, t, *J*2.2Hz, H4), 6.68 (2H, d, *J*2.2Hz, H2, H6), 7.05 (1H, d, *J*16.2Hz, -C = C-H), 7.13 (1H, d, *J*16.2Hz, -C = C-H), 7.59 (2H, d, *J*8.3Hz, H2′, H6′), 7.64 (2H, d, *J*8.3Hz, H3′, H5′), ^13^C NMR (100 MHz, CDCl_3_) δ (ppm) = 55.4 (OMe), 100.9 (C4), 105.1 (C2, C6), 110.7 (C4′), 119.0 (CN), 126.9 (C2′, C6′), 127.2 (C = C), 132.4 (C = C), 132.6 (C3′, C5′), 138.3 (C1′), 141.7 (C1), 161.1 (C3, C5). HRMS calculated *m/z* 288.09950 [(M + Na)]^+^, found 288.12135.

#### (*E*)-1,3-dimethoxy-5-(4-methoxystyryl)benzene (12)

Procedure B gave, after purification, 403 mg white solid (60 %), m.p. = 59–61 °C (literature m.p. = 55–58 °C, [21]), Rf (10:90::ethyl acetate:petroleum ether) = 0.48, IR ν (cm^-1^) = 2960, 2912, 2837, 1605, 1517, 1442, 1239, 965, 824, ^1^H NMR (400 MHz, CDCl_3_) δ (ppm) = 3.83 (9H, s, OMe), 6.38 (1H, t, *J*2.0Hz, H4), 6.65 (2H, d, *J*2.0Hz, H2, H6), 6.89 (2H, d, *J*8.8Hz, H2′, H6′), 6.90 (1H, d, *J*16.2Hz, -C = C-H), 7.04 (1H, d, *J*16.2Hz, -C = C-H), 7.44 (d, *J*8.8Hz, H3′, H5′), ^13^C NMR (100 MHz, CDCl_3_) δ(ppm) = 55.3 (OMe), 55.4 (2xOMe), 99.7 (C4), 104.4 (C2, C6), 114.2 (C2′, C6′), 126.6 (C = C), 127.8 (C3′,C5′), 128.8 (C = C), 130.0 (C1′), 139.7 (C1), 159.4 (C4′), 161.0 (C3, C5). HRMS calculated *m/z* 293.11482 [(M + Na)]^+^, found 293.09290.

#### Methyl (*E*)- 4-(3,5-dimethoxystyryl)benzoate (13)

Procedure A gave 384 mg white solid (59 %), m.p. = 121–122 °C (literature m.p. = 117–121 °C, [22]), Rf (10:90::ethyl acetate:petroleum ether) = 0.51, IR ν (cm^-1^) = 3080, 2999, 2945, 1708, 1587, 1433, 1273, 1152, 966, 697, ^1^H NMR (400 MHz, CDCl_3_) δ (ppm) = 3.83 (6H, s, OMe), 3.92 (3H, s, COOMe), 6.43 (1H, t, *J*2.2Hz, H4), 6.69 (2H, d, *J*2.2Hz, H2, H6), 7.08 (1H, d, *J*16.4Hz, -C = C-H), 7.14 (1H, d, *J*16.4Hz, -C = C-H), 7.55 (2H, d, *J*8.3Hz, H2′, H6′), 8.02 (d, *J*8.2Hz, H3′, H5′), ^13^C NMR (100 MHz, CDCl_3_) δ (ppm) = 52.1 (COO*Me*), 55.4 (OMe), 100.6 (C4), 104.9 (C2, C6), 126.4 (C2′, C6′), 128.1 (C = C), 129.0 (C1′), 130.03 (C3′,C5′), 131.2 (C = C), 138.8 (C1), 141.65 (C4′), 161.1 (C3, C5), 166.8 (*C*OOMe). HRMS calculated *m/z* 321.10973 [(M + Na)]^+^ and 619.23024 [(2 M + Na)]^+^, found 321.10598 and 619.22537.

#### (*E*)-1,3-dimethoxy-5-(4-nitrostyryl)benzene (14)

Procedure A gave 594 mg yellow solid (90 %), m.p. = 137–138 °C (literature m.p. = 135–137 °C, [23]), Rf (10:90::ethyl acetate:petroleum ether) = 0.48, IR ν (cm^-1^) = 3051, 2839, 1636, 1592, 1503, 1329, 1295,1205, 1106, 948, 824, ^1^H NMR (400 MHz, CDCl_3_) δ (ppm) = 3.86 (6H, s, OMe), 6.47 (1H, t, *J*2.4Hz, H4), 6.71 (2H, d, *J*2.4Hz, H2, H6), 7.11 (1H, d, *J*18.0Hz, -C = C-H), 7.20 (1H, d, *J*18.0Hz, -C = C-H), 7.63 (2H, d, *J*9.7Hz, H2′, H6′), 8.22 (2H, d, *J*9.7Hz, H3′, H5′), ^13^C NMR(100 MHz, CDCl_3_) δ(ppm) = 55.4 (OMe), 101.0 (C4), 105.12 (C2, C6), 124.1 (C3′, C5′), 126.8 (C = C), 126.9 (C2′,C6′), 133.3 (C = C), 138.1 (C1′), 143.7 (C1), 146.8 (C4′), 161.1 (C3, C5). HRMS calculated *m/z* 308.08933 [(M + Na)]^+^, found 308.08817.

#### (*E*)-2-(3,5-dimethoxystyryl)-1,3-difluorobenzene (15)

Procedure A gave 520 mg shiny white crystals (78 %), m.p. 85–86 °C, Rf (10:90::ethyl acetate:petroleum ether) = 0.64, IR ν (cm^-1^) = 3009, 2973, 2844, 1589, 1463, 1306, 1200, 1152, 979, 788, 682, ^1^H NMR (400 MHz, CDCl_3_) δ (ppm) = 3.83 (6H, s, OMe), 6.43 (1H, t, *J*2.2Hz, H4), 6.69 (2H, d, *J*2.2Hz, H2, H6), 6.92 (2H, t, *J*8.4Hz, H3′, H5′), 7.10 (d, *J*16.8Hz, -C = C-H) 7.15 (1H, tt, *J*8.4, 8.4Hz, H4′), 7.37 (1H, d, *J*16.4Hz, -C = C-H), ^13^C NMR (100 MHz, CDCl_3_) δ (ppm) = 55.4 (OMe), 100.6 (C4), 104.7 (C2, C6), 111.7 (C3′, C5′, *J*_*C-F*_6.47), 114.7 (C1′, *J*_*C-F*_15.1Hz), 115.7 (C = C), 128.0 (C4′, *J*_*C-F*_10.7Hz), 135.1 (C = C, *J*_*C-F*_8.35 Hz), 139.6 (C1), 160.9 (C2′, C6′, *J*_*C-F*_250, 7.7 Hz), 161.0 (C3, C5). HRMS calculated *m/z* 299.08541 [(M + Na)]^+^, found 299.10582.

#### (*E*)-1,3-dichloro-2-(3,5-dimethoxystyryl)benzene (16)

Procedure B gave 508 mg, white solid (79 %), m.p. 66–67 °C, Rf (10:90::ethyl acetate:petroleum ether) = 0.64, IR ν (cm^-1^) = 2959, 2897, 2833, 1598, 1424, 1348, 1206, 1152, 1054, 965, 819, 763, 676, ^1^H NMR (400 MHz, d_6_-DMSO) δ (ppm) = 3.78 (6H, s, OMe), 6.48 (1H, t, *J*2.0Hz, H4), 6.78 (2H, d, *J*2.0Hz, H2, H6), 6.99 (1H, d, *J*16.4Hz, -C = C-H), 7.15 (1H, d, *J*16.4Hz, -C = C-H), 7.32 (1H, t, *J*8.0Hz, H4′), 7.52 (2H, d, *J*8.0Hz, H3′, H5′), ^13^C NMR (100 MHz, d_6_-DMSO) δ (ppm) = 55.3 (OMe), 100.9 (C4), 104.7 (C2, C6), 122.7 (C = C), 128.8 (C3′, C5′), 129.3 (C4′), 133.6 (C2′, C6′), 134.1 (C1’), 136.8 (C = C), 138.1 (C1), 160.7 (C3, C5). HRMS calculated *m/z* 331.02631 [(M + Na)]^+^ and 641.06044 [(2 M + Na)]^+^, found m/z 331.04468 and 641.09081.
